# High Expression of DLGAP5 Indicates Poor Prognosis and Immunotherapy in Lung Adenocarcinoma and Promotes Proliferation through Regulation of the Cell Cycle

**DOI:** 10.1155/2023/9292536

**Published:** 2023-01-19

**Authors:** Xiaolong Tang, Honghong Zhou, Yongshuo Liu

**Affiliations:** ^1^Department of Clinical Laboratory Diagnostics, Binzhou Medical University, Binzhou, Shandong 256603, China; ^2^Key Laboratory of RNA Biology, Center for Big Data Research in Health, Institute of Biophysics, Chinese Academy of Sciences, Beijing 100101, China; ^3^Department of Clinical Laboratory, Binzhou Medical University Hospital, Binzhou, Shandong 256603, China; ^4^Biomedical Pioneering Innovation Center (BIOPIC), Beijing Advanced Innovation Center for Genomics, Peking-Tsinghua Center for Life Sciences, Peking University Genome Editing Research Center, State Key Laboratory of Protein and Plant Gene Research, School of Life Sciences, Peking University, Beijing 100871, China

## Abstract

**Background:**

Lung adenocarcinoma (LUAD) is one of the most common types of cancer in the respiratory system, with a high mortality and recurrence rate. The role of disc large-associated protein 5 (DLGAP5) in LUAD progression and tumor microenvironment (TME) remains unclear. This study is aimed at revealing the functional role of DLGAP5 in LUAD based on bioinformatics analysis and experimental validation.

**Methods:**

Differential expression analysis, protein-protein interaction (PPI) network, and Cox regression analysis were applied to screen potential prognostic biomarkers. The mRNA and protein levels of DLGAP5 were analyzed using The Cancer Genome Atlas (TCGA) and the Human Protein Atlas (HPA) databases. The CCK-8 and colony formation assays were performed to assess the effect of DLGAP5 on cell proliferation. RNA sequencing (RNA-seq) and enrichment analyses were utilized to explore the biological functions of DLGAP5. Furthermore, flow cytometry was used to explore the role of DLGAP5 on the cell cycle. The ssGSEA algorithm in the R package “GSVA” was applied to quantify immune infiltrating cells, and the tumor immune dysfunction and exclusion (TIDE) algorithm was used to predict the efficacy of immunotherapy. Moreover, analyses using the cBioPortal and MethSurv databases were performed to evaluate the mutation and methylation of DLGAP5, respectively. Finally, the prognostic value of DLGAP5 was estimated using the TCGA and the Gene Expression Omnibus (GEO) databases. The nomogram model was constructed using the TCGA-LUAD cohort and evaluated by adopting calibration curves, time-dependent receiver operating characteristic (ROC) curves, and decision curve analysis (DCA).

**Results:**

DLGAP5 mRNA and protein abundance were significantly elevated in LUAD, and knockdown of DLGAP5 remarkably suppressed lung cancer cell proliferation through induction of cell cycle G1 arrest. In addition, DLGAP5 expression was positively correlated with Th2 cells and negatively correlated with B cells, T follicular helper cells, and mast cells. LUAD patients with high DLGAP5 expression may be resistant to immunotherapy. Hypermethylation levels of the cg23678254 site of DLGAP5 or its enhanced expression were unfavorable for the survival of LUAD patients. Meanwhile, DLGAP5 expression was associated with TNM stages, tumor status, and therapy outcome. Notably, the prognostic model constructed based on DLGAP5 expression exhibited great predictive capability, which was promising for clinical applications.

**Conclusion:**

DLGAP5 promotes lung cancer cell proliferation through regulation of the cell cycle and is associated with multiple immune infiltrating cells. Furthermore, DLGAP5 predicts poor prognosis and response to immunotherapy in lung adenocarcinoma.

## 1. Introduction

Lung cancer is the predominant cause of cancer-related deaths worldwide and is divided approximately into small-cell lung cancer (SCLC, approx. 15% cases) and non-small-cell lung cancer (NSCLC, approx. 85% cases), with the major histological subtypes of NSCLC being adenocarcinoma and squamous cell carcinoma [1]. LUAD generally evolves from mucosal glands and accounts for approximately 40% of all lung cancers. In most cases, it may be discovered in areas of scarring or chronic inflammation around the lungs [2]. Unfortunately, LUAD remains one of the most aggressive and rapidly fatal types of cancer, with overall survival (OS) of less than 5 years for LUAD patients [3]. With advances in medical technology, LUAD patients are benefiting from immunotherapy in addition to conventional surgical resection and chemoradiotherapy [4]. Immune checkpoint inhibitors (ICIs) have been extensively studied and applied to LUAD patients with promising outcomes [5, 6]. Although immunotherapy has brought unprecedented survival benefits, the efficacy is much better in LUAD patients with high programmed cell death ligand 1 (PD-L1) expression [7]. As a result, overall cure and survival rates remain low, especially when tumors metastasize [8]. Identification of new molecular targets in LAUD remains the grand central question in the clinical intervention of the disease.

The disc large-associated protein (DLGAP) family members are originally detected in rats and compose of DLGAP1, 2, 3, 4, and 5, respectively [9]. DLGAP1 to 4 hold a role as scaffolding proteins in postsynaptic density and are directly implicated in various psychological and neurological disorders [9]. DLGAP5 is also known as KIAA0008, disc large homolog 7 (DLG7), and hepatoma upregulated protein (HURP). The mRNA transcript of DLGAP5 is expressed at S-phase and maintained at both G2- and M-phases [10]. Physiologically, DLGAP5 has a unique function in maintaining microtubule growth and stability in the spindle by promoting microtubule polymerization and bipolar spindle formation [11, 12]. Furthermore, DLGAP5 knockout in mice causes female infertility, but DLGAP5 is dispensable for normal mouse development [13]. Another study also shows that uterine expression of DLGAP5 may be linked to female reproductive function during the menstrual cycle [14]. DLGAP5 plays an important role in tumorigenesis, metastasis, and drug resistance as well. Specifically, the knockdown of DLGAP5 not only significantly inhibited the proliferation and invasion of colorectal, clear cell renal cell carcinoma, hepatocellular carcinoma, and pancreatic cancer cells but also induced cell cycle arrest in ovarian and breast cancer cells [15–20]. Strikingly, elevated DLGAP5 expression suppressed apoptosis in prostate cancer and hepatocellular carcinoma cells induced by *γ*-radiation and cisplatin, respectively [21, 22]. Meanwhile, as a prognostic biomarker, DLGAP5 was associated with poor prognosis in colorectal, endometrial, breast, and pancreatic cancers [15, 18, 20, 23]. Overall, DLGAP5 is a promising target for antitumor therapy.

Currently, the role of DLGAP5 has been rarely reported in LUAD. This study is aimed at comprehensively investigating the potential functions of DLGAP5. First, we identified DLGAP5 as a potential oncogene by differential expression analysis, PPI network, and Cox regression analysis. Next, we comprehensively evaluated the relationship between DLGAP5 expression and cell proliferation, cell cycle, immune infiltration, immunotherapy efficacy, and prognosis. Finally, we further elucidated the relationship between DLGAP5 expression and clinical stages of LUAD patients and constructed a prognostic model with great predictive capability.

## 2. Material and Method

### 2.1. Database and Data Processing

First, we screened for genes highly expressed in LUAD tissues using four independent cohorts in the GEO database (http://www.ncbi.nlm.nih.gov/geo/), namely, the GSE7670, GSE43458, GSE116959, and GSE140797 datasets. GSE143423 was applied to assess the expression of DLGAP5 at the single-cell level. In addition, GSE31210 and GSE50081 were used as validation cohorts for survival analysis. Detailed information is shown in Table 1.

Gene expression data and corresponding clinical information from LUAD patients in the TCGA database (https://portal.gdc.cancer.gov/) were utilized for subsequent analysis, including 59 normal and 535 LUAD tissues. The data format HTSeq-Counts (high-throughput sequencing-counts) was applied for differential expression analysis to classify patients in the TCGA-LUAD cohort into two groups based on the median DLGAP5 expression. Next, the HTSeq-FPKM (fragments per kilobase of transcript per million fragments mapped) data format was converted into TPM (transcripts per million) data format for subsequent analysis. All analyses were preceded by a log 2 transformation of all RNA-seq data. Unavailable or unknown clinical features were considered missing values.

### 2.2. Screening for LUAD Oncogenes

Grouping by LUAD and normal lung tissues, the GSE7670, GSE43458, GSE116959, and GSE140797 datasets were subjected to differential expression analysis by the online tool GEO2R (https://www.ncbi.nlm.nih.gov/geo/geo2r/) [24], respectively, to obtain the differentially expressed gene (DEG) matrices and draw volcano plots using “ggplot2” with a threshold value of |log *FC*| ≥ 1 and adjust *P* < 0.05. The Venn diagrams take the intersection of all up- and downregulated genes in the four datasets. Next, we extracted the overlapping up- and downregulated DEGs and utilized the STRING (https://www.string-db.org/) [25] online tool to construct a PPI network with interaction scores greater than 0.700. We then imported the data into Cytoscape software (http://www.cytoscape.org; version 3.8.0) [26] for graphical optimization and used the molecular complex detection (MCODE) application in Cytoscape software to identify highly connected DEGs. Finally, univariate and multivariate Cox regression analyses were further performed to screen genes associated with prognosis using the R package “survival,” and forest plots were visualized using “ggplot2.”

### 2.3. DLGAP5 mRNA and Protein Expression Levels

First, the RNA-seq data of DLGAP5 in each tumor and normal tissue were obtained from the TCGA and the Genotype-Tissue Expression (GTEx) databases by UCSC XENA (https://xena.ucsc.edu/). The ROC curve was used to detect the predictive accuracy of DLGAP5 in LUAD and normal lung tissue using the TCGA-LUAD cohort, analyzed with the R package “pROC.” The TCGA-LUAD cohort was used to examine the differential expression of DLGAP5 mRNA levels in LUAD and normal lung tissues. Immunohistochemical images of DLGAP5 in LUAD and normal lung tissues were downloaded from the HPA database (https://www.proteinatlas.org/).

### 2.4. Exploration of DLGAP5 Pathways

First, 535 LUAD patients in the TCGA-LUAD cohort were divided into two groups according to the median DLGAP5 expression and subjected to differential expression analysis using the R package “DESeq2” with a threshold of |log *FC*| ≥ 1.5 and adjust *P* < 0.05. Then, DLGAP5-related genes were subjected to Gene Ontology (GO) and gene set enrichment analysis (GSEA) using the R package “clusterProfiler,” and the R packages “ggplot2” and “enrichplot” were used for visualization.

### 2.5. Role of DLGAP5 in the TME and Immunotherapy

First, the level of immune cell infiltration in the TME was quantified by the ssGSEA algorithm in the R package “GSVA,” in which markers for 24 immune cell types were referenced from the paper published by Bindea et al. [27]. Subsequently, correlation analysis was performed to analyze DLGAP5 expression with immune cells and immunosuppressive checkpoints using the TCGA-LUAD cohort. Data on immune subtypes of LUAD patients in TCGA were obtained from the paper published by Thorsson et al. [28]. Finally, we adopted TIDE algorithm (http://tide.dfci.harvard.edu/) to predict the immunotherapy response in LUAD patients in the DLGAP5-high and DLGAP5-low groups. LUAD patients with high TIDE scores showed a poor response to immunotherapy [29].

### 2.6. Mutation Analysis and Methylation Analysis of DLGAP5

The cBioPortal web tool (http://www.cbioportal.org/) was used to analyze the mutation rate of DLGAP5 in LUAD patients and the association with prognosis. This study utilized nine separate lung adenocarcinoma datasets, which included 3299 patients and 3394 specimens. Five of these datasets contained DLGAP5 mutation data.

The MethSurv database (https://biit.cs.ut.ee/methsurv/) was applied to evaluate the DNA methylation sites of DLGAP5 in LUAD patients and to further investigate its prognostic value.

### 2.7. Prognostic Value and Predictive Efficacy of DLGAP5

We collected clinical data from 535 patients in the TCGA-LUAD cohort, including pathologic stage, TNM stage, residual tumor, tumor status, therapy outcome, and gender. Next, these clinicopathological characteristics were subjected to multigroup survival analyses using the R package “survival,” and the R package “survminer” was used for visualization. We also evaluated the expression of DLGAP5 in various clinicopathological characteristics.

Subsequently, TCGA-LUAD (*n* = 526), GSE31210 (*n* = 226), and GSE50081 (*n* = 127) were utilized to validate the prognostic value of DLGAP5 in patients with LUAD. The Kaplan-Meier survival analyses were conducted using the R package “survival” and visualized using the R package “survminer.” Time-dependent ROC curves were applied to evaluate the accuracy of DLGAP5 in predicting overall survival in LUAD patients, and the R package “timeROC” was used for analysis.

Finally, univariate and multivariate Cox regression analyses were performed to screen risk factors in patients with LUAD. Next, DLGAP5 expression with partial clinicopathological characteristics was utilized to construct a nomogram and plot calibration curves using the R package “rms.” Time-dependent ROC curves and DCA were applied to assess the predictive capability of the nomogram model using the R packages “timeROC,” “survival,” and stdca R [30], respectively.

### 2.8. Cell Culture and Lentiviral Packaging and Infection

All cell lines were purchased from the American Type Culture Collection (ATCC) and stored at the Cancer Hospital of the Chinese Academy of Medical Sciences. All cell lines were identified by short tandem repeat (STR) profiling. Lung cancer cell lines A549 and H1975 were cultured in the Dulbecco's Modified Eagle Medium (DMEM, A549) (Gibco, USA) and Roswell Park Memorial Institute 1640 (RPMI 1640, H1975) (Gibco, USA) medium containing 10% fetal bovine serum (FBS, BI). HEK293T cells were cultured in DMEM medium containing 10% fetal bovine serum and penicillin (100 units/ml)-streptomycin (100 mg/ml). All cells were cultured at 37°C in a constant temperature incubator containing 5% CO_2_.

HEK293T cells were used for lentiviral production. Lentiviral expression vector pLKO was used to construct the DLGAP5 knockdown vector (shDLGAP5). During preparation, 500 ng of target gene plasmid was added into 100 *μ*l opti-MEM together with 50 ng VSVG, 500 ng pR8.74, and 3 *μ*l transfection reagent PEI, mixed sufficiently, and left for 15 minutes, and then, the mixture was added into approximately 80% confluent HEK293T cells in 12-well plates. The supernatant containing lentivirus was harvested 72 hours after transfection, filtered through a 0.45 mM PES filter, and then stored at -80°C for backup. Subsequently, A549 and H1975 cells were seeded in six-well plates, and 24 hours later, 50 *μ*l of viral solution and polybrene (1 : 1000) was added. 24 hours after infection, the cell culture medium containing viral solution was replaced with fresh complete cell culture medium with puromycin.

The target sequences of the shDLGAP5 included the following:

shDLGAP5-1: CCGGGCATTCCACAACAAACTACATCTCGAGATGTAGTTTGTTGTGGAATGCTTTTTG

shDLGAP5-2: CCGGGCACAGCAGTTGGTCAAACAACTCGAGTTGTTTGACCAACTGCTGTGCTTTTTG

shDLGAP5-3: CCGGCGAGAGTGATGTTCGAGCAATCTCGAGATTGCTCGAACATCACTCTCGTTTTTG

shDLGAP5-4: CCGGCATAAGGAATACGAACGAAATCTCGAGATTTCGTTCGTATTCCTTATGTTTTTG

### 2.9. Western Blotting and Reverse Transcription and Quantitative Real-Time PCR (RT-qPCR)

Cells were collected in 1.5 ml EP tubes, RIPA lysis buffer containing protease inhibitors and phosphatase inhibitors was added, and cells were lysed sufficiently to obtain cellular proteins. Protein concentrations were determined using the BCA Protein Concentration Assay Kit (Thermo Fisher, Waltham, MA, USA) according to the manufacturer's instructions. Equal amounts of proteins were separated in 12% SDS-PAGE; then, proteins were transferred to PVDF membranes, blocked with 5% skim milk powder for 1 hour at room temperature, and then incubated with a 1 : 1000 dilution of protein primary antibody at 4°C overnight. The membranes were washed three times with TBST, then incubated with fluorescent secondary antibody for 1 hour at room temperature protected from light, and then washed three more times with TBST. Antibodies against DLGAP5 (12038-1-AP, Proteintech) and GAPDH (60004-1-Ig, Proteintech) were used.

According to the manufacturer's protocol, total RNA was extracted using an RNA-easy Isolation Reagent (Vazyme, China). RNA concentration was quantified using NanoDrop ND2000 (Thermo Fisher, Waltham, MA, USA). 1 *μ*g of RNA per sample was reverse transcribed into cDNA using the Tiangen Reverse Transcription Kit, and the cDNA products were diluted to a final concentration of 10 ng/*μ*l. Real-time PCR was performed using 2× SYBR Green Premix Ex Taq (Takara, Shiga, Japan) on an ABI 7500 PCR system (Applied Biosystems, CA, USA). Primer pairs are listed below. Analyses were performed using the comparative cycle threshold (CT) method, and all samples were normalized to GAPDH expression. The sequences of primers used were as follows:

GAPDH forward: GGAGCGAGATCCCTCCAAAAT

GAPDH reverse: GGCTGTTGTCATACTTCTCATGG

DLGAP5 forward: AAGTGGGTCGTTATAGACCTGA

DLGAP5 reverse: TGCTCGAACATCACTCTCGTTAT

### 2.10. Cell Proliferation Assay

The 96-well plates were seeded with 2 × 10^3^ cells per well and incubated in an incubator at 37°C with 5% CO_2_. Then, 10 *μ*l of CCK-8 solution was added to each well, and the absorbance at 450 nm was measured using an enzyme marker after 0, 24, 48, 72, and 96 hours, respectively. In addition, cells were seeded at 2 × 10^3^ cells per well in six-well plates and incubated for ten days at 37°C with 5% CO_2_. The number of colony formations was counted and photographed.

### 2.11. Bulk RNA-seq Analysis

Total RNA was extracted from A549 shCtrl and A549 shDLGAP5 and then subjected to PE150 Hiseq, performed by Novogene (Beijing, China). Each sample contained pooled RNAs from 3 biological replicates. Gene expression levels were quantified by a software package called RSEM. Significance lists were manipulated by setting a threshold of |log *FC*| ≥ 1 and adjust *P* < 0.05 using R package “DEseq2.” The resulting list of all differentially expressed genes was subsequently analyzed for the enrichment of biological themes using the DAVID bioinformatics platform.

### 2.12. Cell Cycle Assay

First, the collected cell samples were fixed with cold alcohol. Before cell staining, all fixative was removed from the cells. Then, the sample cell concentration was adjusted to 1 × 10^6^ cells/ml using phosphate-buffered saline. Each sample contained 1 ml of cell suspension, and the permeability reagent Triton X-100 and 1 *μ*l FxCycle Violet stain were added and mixed well. The samples were incubated for 30 minutes at room temperature protected from light. Finally, samples were analyzed in a flow cytometer without washing, using 405 nm excitation and emission collected at 450/50 bandpass or equivalent.

### 2.13. Statistical Analysis

All statistical analyses were processed on R Studio software (https://www.rstudio.com/; version 4.1.1), and *P* value < 0.05 was considered statistically significant. In this study, *t* Welch, *t* Student, and Wilcoxon rank sum test were used for comparison between groups. Spearman's test was performed for all correlation analyses. Cox regression and log-rank test were applied for survival analysis.

## 3. Results

### 3.1. Screening for Key Oncogenes in LUAD

**Figure 1:**
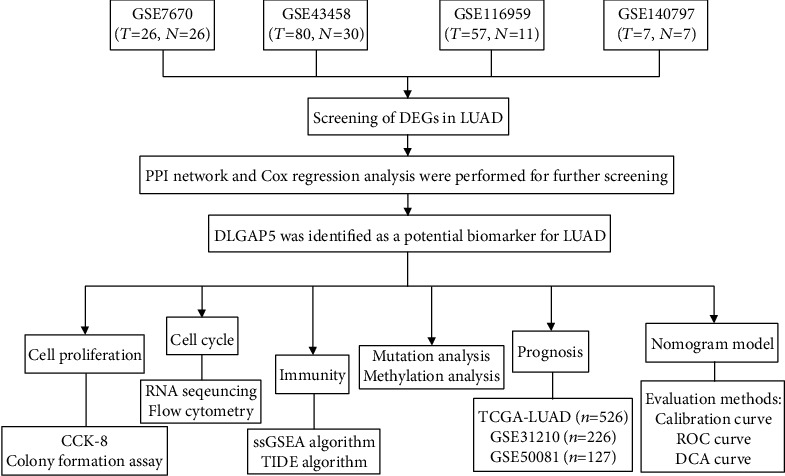
Flow chart of this study.

**Figure 2:**
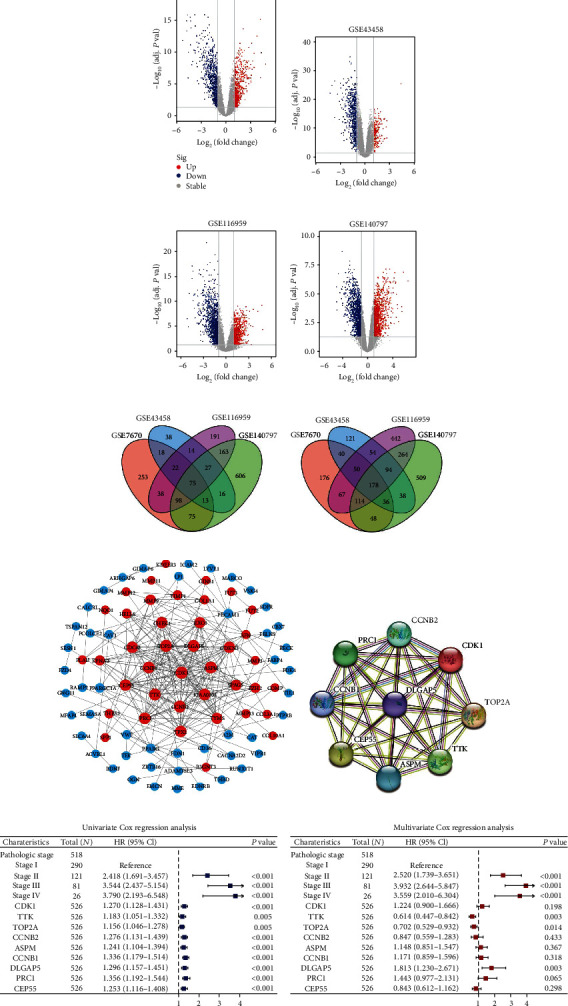
Screening of oncogenes in LUAD. (a–d) Volcano plots of differential gene expression matrices for GSE7670, GSE43458, GSE116959, and GSE140797, respectively. (e, f) Venn plots of up- and downregulated overlapping DEGs. (g) The overlapping DEGs constructed a PPI network. Red nodes indicated the upregulated DEGs, and blue nodes represented the downregulated DEGs. (h) Highly connected DEGs were extracted and reconstructed as a PPI network using the STRING online database. (i, j) Univariate and multivariate Cox regression analyses were performed to further screen LUAD for key oncogene using the TCGA database.

The overview of the process used in our study is shown in Figure 1. In total, 593 upregulated and 709 downregulated genes were filtered from GSE7670 (Figure 2(a)), 223 upregulated and 611 downregulated genes from GSE43458 (Figure 2(b)), 628 upregulated and 1263 downregulated genes from GSE116959 (Figure 2(c)), and 1073 upregulated and 1281 downregulated genes from GSE140797 (Figure 2(d)). Ultimately, 75 overlapping upregulated genes (Figure 2(e)) and 178 overlapping downregulated genes (Figure 2(f)) were extracted from the LUAD group compared with the control group. Subsequently, these DEGs constructed a PPI network containing 88 nodes and 244 edges by setting the interaction score as high confidence (0.700), with 40 upregulated genes and 50 downregulated genes included (Figure 2(g)). In which, highly connected DEGs were extracted and reconstructed a PPI network, namely, CDK1, TTK, TOP2A, CCNB2, ASPM, CCNB1, DLGAP5, PRC1, and CEP55 (Figure 2(h)). Subsequently, the above candidate DEGs were further subjected to univariate and multivariate Cox regression analyses based on the TCGA-LUAD cohort, suggesting that DLGAP5 may be an independent prognostic factor in LUAD (Figures 2(i) and 2(j)).

### 3.2. DLGAP5 Was Highly Expressed and Promoted the Proliferation of Lung Cancer Cells

**Figure 3:**
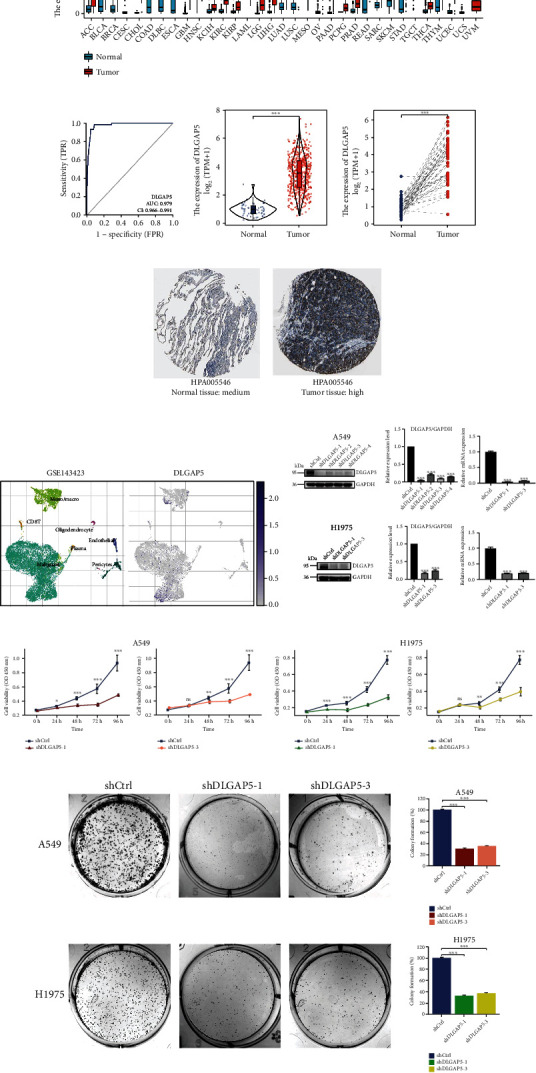
DLGAP5 was highly expressed in LUAD and promoted cell proliferation. (a) Comparison of DLGAP5 expression in tumor and normal tissues in pan-cancer using TCGA and GTEx databases. (b) ROC was utilized to examine the predictive accuracy of DLGAP5 expression on LUAD tissue. (c, d) Differential mRNA expression levels of DLGAP5 in unpaired and paired LUAD specimens, respectively. (e, f) Differential protein expression of DLGAP5 in normal and LUAD tissues. (g) Differences in DLGAP5 expression at the single-cell level. (h) Western blot and RT-qPCR were applied to verify the knockdown efficiency of DLGAP5 in A549 and H1975 cell lines. (i, j) CCK-8 assay was utilized to examine the effect of DLGAP5 knockdown on the proliferative capacity of A549 and H1975 cells, respectively. (k) Colony formation assays of knockdown DLGAP5 in A549 and H1975 cell lines, respectively. Significance codes: ns: not significant. ^∗^*P* < 0.05, ^∗∗^*P* < 0.01, and ^∗∗∗^*P* < 0.001.

Excluding mesothelioma (MESO) and uveal melanoma (UVM) without corresponding paraneoplastic tissue specimens, the pan-cancer analysis revealed that significant upregulation of DLGAP5 in 30 of 31 cancers compared to paraneoplastic tissue, but downregulation in acute myelogenous leukemia (LAML) (Figure 3(a)). To clarify the specific expression of DLGAP5 in LUAD tissues, the ROC curve revealed that the area under the curve (AUC) of DLGAP5 is 0.976, which displayed an extremely high accuracy (Figure 3(b)). DLGAP5 mRNA expression was significantly upregulated in LUAD tissues compared to paraneoplastic tissues, both in unpaired and paired samples (Figures 3(c) and 3(d)). Likewise, immunohistochemical staining also indicated that DLGAP5 protein expression was upregulated in LUAD tissues (Figures 3(e) and 3(f)). Single-cell profiles revealed that DLGAP5 was predominantly expressed in malignant cells, followed by monocytes/macrophages (Figure 3(g)).

Furthermore, we further verified whether the abnormally elevated DLGAP5 expression was related to the proliferation of lung cancer cells. First, we verified the knockdown efficiency of DLGAP5 in A549 and H1975 cell lines by Western blot and RT-qPCR, suggesting the selection of shDLGAP5-1 and shDLGAP5-3 for subsequent experiments (Figure 3(h)). The CCK-8 assay revealed that DLGAP5 knockdown suppressed the proliferation capability of A549 and H1975 cells (Figures 3(i) and 3(j)). In addition, the number of colonies was remarkably decreased in the DLGAP5 knockdown group by comparison with the control group (Figure 3(k)). Thus, DLGAP5 was highly expressed in LUAD tissues and promoted cell proliferation.

### 3.3. DLGAP5 Was Implicated in the Regulation of the Cell Cycle

**Figure 4:**
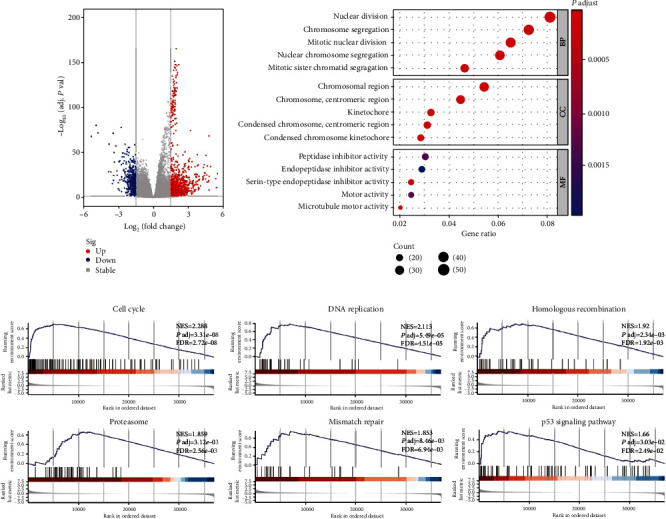
Investigation of the biological functions of DLGAP5 using TCGA-LUAD cohort. (a) Volcano plot showing DLGAP5-related DEGs. (b) GO enrichment analysis for DLGAP5-related DEGs. BP: biological processes; CC: cellular component; MF: molecular function. (c) GSEA for DLGAP5-related DEGs.

First, the 535 LUAD patients in the TCGA database were grouped according to the median DLGAP5 expression and subjected to differential expression analysis with a threshold of |log *FC*| ≥ 1.5 and adjust *P* < 0.05. In total, 1078 upregulated genes and 459 downregulated genes were obtained (Figure 4(a)). GO enrichment analysis indicated that DLGAP5-related genes were primarily implicated in biological processes such as nuclear division, chromosome segregation, and mitotic nuclear division. Transcription proteins were mostly located in the chromosomal, centromeric region, and kinetochore. Molecular functions were mostly concentrated in peptidase inhibitor activity, serine-type endopeptidase inhibitor activity, and motor activity (Figure 4(b)). GSEA revealed that DLGAP5-related genes were mainly associated with cell cycle-related pathways, such as DNA replication, homologous recombination, proteasome, mismatch repair, and p53 signaling pathway (Figure 4(c)).

**Figure 5:**
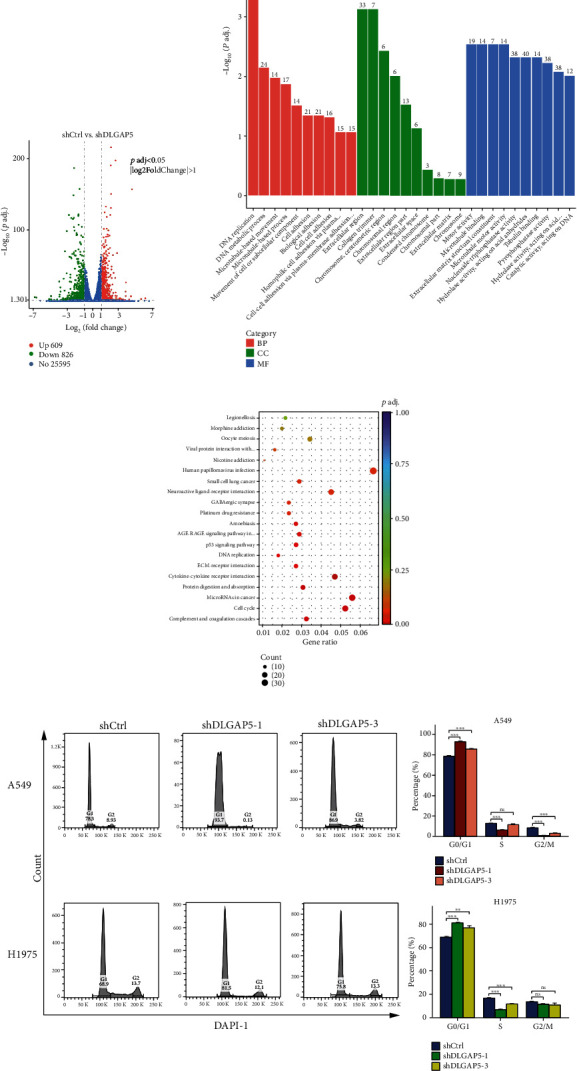
DLGAP5 was involved in the regulation of the cell cycle. (a) Volcano plot of DLGAP5-related DEGs in the shCtrl group compared with the shDLGAP5 group. (b, c) GO and KEGG enrichment analyses for DLGAP5-related DEGs in the shCtrl group compared with the shDLGAP5 group. (d) Flow cytometry was utilized to examine the effect of knockdown of DLGAP5 on the cell cycle of A549 and H1975 cells. Significance codes: ns: not significant. ^∗^*P* < 0.05, ^∗∗^*P* < 0.01, and ^∗∗∗^*P* < 0.001.

To further confirm the reliability of the DLGAP5-related pathways, we performed RNA sequencing and differential expression analysis using the shCtrl group and shDLGAP5 group in A549 cell lines with a threshold of |log *FC*| ≥ 1 and adjust *P* < 0.05. In total, 609 upregulated genes and 826 downregulated genes were identified (Figure 5(a)). Consistent with the previous results, DLGAP5-related genes were mainly involved in cell cycle regulation (Figures 5(b) and 5(c)). Next, the knockdown of DLGAP5 in A549 and H1975 cell lines significantly induced cell cycle G1 arrest (Figure 5(d)). Collectively, these data strongly demonstrated that DLGAP5 promotes the proliferation of lung cancer cells by regulating the cell cycle.

### 3.4. Role of DLGAP5 in the TME and Immunotherapy

**Figure 6:**
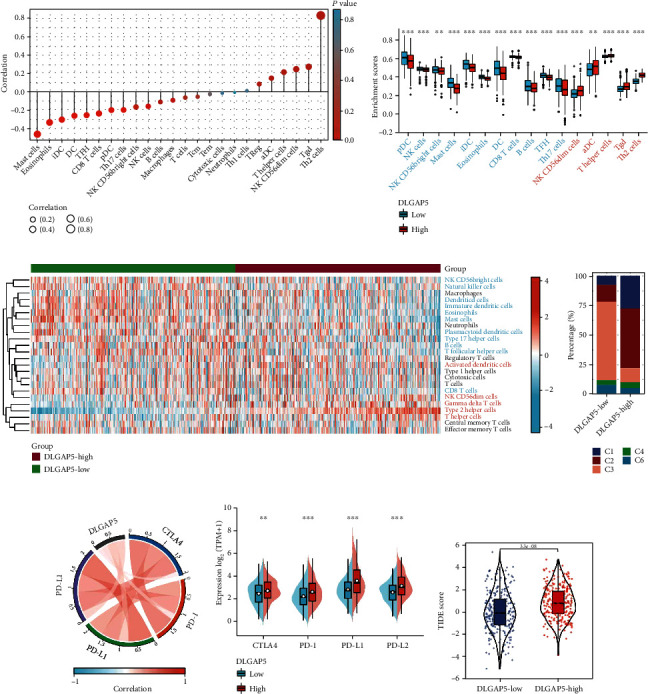
Role of DLGAP5 in the TME and immunotherapy. (a) Correlation of DLGAP5 expression with 24 types of immune cells. (b) Differences in the enrichment scores of 24 immune cell types between the DLGAP5-high and DLGAP5-low groups. (c) Heat map showing the difference in the infiltration abundance of immune cells in the DLGAP5-high and DLGAP5-low groups. Red font indicated that immune cells were infiltrated in higher abundance in the DLGAP5-high than in the DLGAP5-low groups. The opposite was true for blue font. (d) Differences in immune subtypes between the DLGAP5-high and DLGAP5-low groups. C1, wound healing; C2, IFN-gamma dominant; C3, inflammatory; C4, lymphocyte depleted; C6, TGF-*β* dominant. (e) Correlation of DLGAP5 expression with four immunosuppressive checkpoints, including CTLA4, PD-1, PD-L1, and PD-L2. (f) CTLA4, PD-1, PD-L1, and PD-L2 were differentially expressed in the DLGAP5-high and DLGAP5-low groups. (g) Differences in TIDE scores in the DLGAP5-high and DLGAP5-low groups. Significance codes: ^∗^*P* < 0.05, ^∗∗^*P* < 0.01, and ^∗∗∗^*P* < 0.001.

Strikingly, the Kyoto Encyclopedia of Genes and Genomes (KEGG) pathway enrichment analysis also revealed that DLGAP5-related genes were related to immune pathways, such as cytokine-cytokine receptor interaction (Figure 5(c)). Therefore, we hypothesized that DLGAP5 holds a unique role in the TME. First, we evaluated the relevance of DLGAP5 expression to immune cells. The result showed that DLGAP5 correlated with a variety of immune cells, especially with Th2 cells in a significant positive correlation (Figure 6(a) and Table 2). In the DLGAP5-high group, the infiltration abundance of Th2 cells, gamma delta T cells, T helper cells, activated dendritic cells, and NK CD56dim cells was higher. In contrast, plasmacytoid dendritic cells, NK cells, NK CD56bright cells, mast cells, immature dendritic cells, eosinophils, dendritic cells, CD8 T cells, B cells, T follicular helper cells, and Th17 cells were more abundant in the DLGAP5-low group (Figures 6(b) and 6(c)). Interestingly, our previous work found that infiltration of B cells, T follicular helper cells, and mast cells favored the prognosis of LUAD patients, while Th2 cells were detrimental [31]. Furthermore, immune subtype C3 had a greater proportion in the DLGAP5-low group, whereas C1 and C2 were less represented than the DLGAP5-high group (Figure 6(d)). Notably, patients with immune subtype C3 had a better prognosis compared to C1 and C2. Overall, these data suggested that DLGAP5 had a specific role in the TME and was associated with prognosis.

Currently, in light of the widespread clinical applications of immune checkpoint inhibitors, we further investigated the relationship between DLGAP5 expression and four immunosuppressive checkpoints, including programmed cell death protein 1 (PD-1), cytotoxic T-lymphocyte-associated protein 4 (CTLA4), PD-L1, and PD-L2. The result was that DLGAP5 was positively correlated with all four immunosuppressive checkpoints (Figure 6(e)). In the DLGAP5-high group, the expression of these four immunosuppressive checkpoints was higher compared to the DLGAP5-low group, especially for PD-L1 (Figure 6(f)). Of note, TIDE scores were higher in the DLGAP5-high group, indicating a poorer immunotherapy efficacy in LUAD patients with high DLGAP5 expression (Figure 6(g)). Collectively, these results suggested that DLGAP5 may facilitate the formation of an immunosuppressive microenvironment where tumor cells evade the surveillance of the immune system, creating appropriate conditions for tumor cell proliferation while remaining unresponsive to immunotherapy.

### 3.5. Effect of DLGAP5 Mutation and Methylation on Survival of LUAD Patients

**Figure 7:**
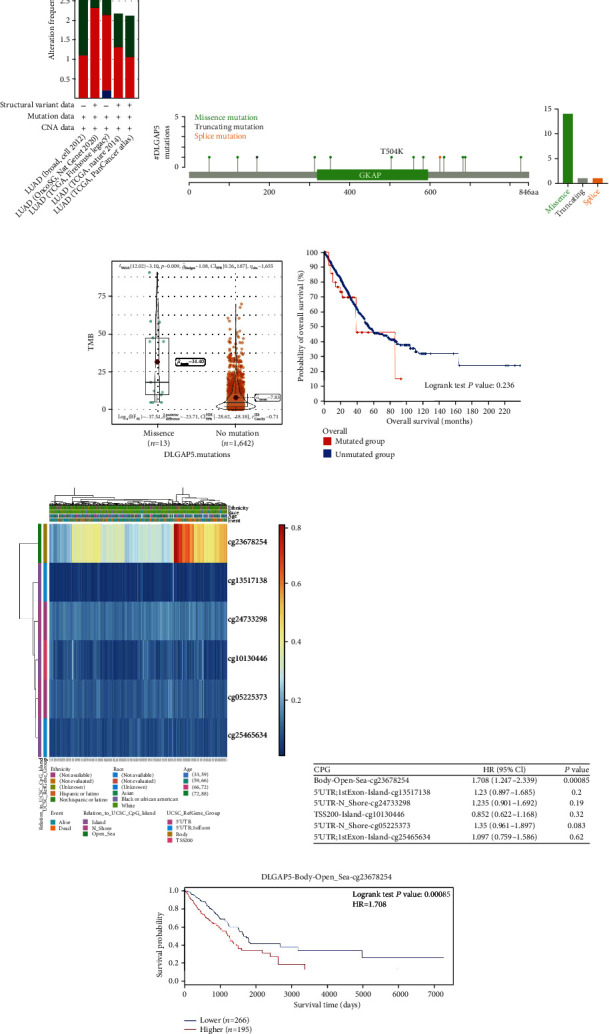
Effect of DLGAP5 mutation and methylation on survival of LUAD patients. (a) The prevalence of DLGAP5 mutations in five independent LUAD cohorts. (b) The subtypes and distributions of PTPN2 somatic mutations. (c) Correlation of DLGAP5 mutations with TMB in LUAD. (d) The Kaplan-Meier curve demonstrated the effect of DLGAP5 mutations on overall survival in LUAD patients. (e) Visualization of DLGAP5 methylation sites in LUAD patients. (f) Impact of different DLGAP5 methylation sites on the prognosis of LUAD patients. (g) The Kaplan-Meier curve showed the effect of DLGAP5 methylation on overall survival in LUAD patients.

To explore the impact of DLGAP5 mutations in LUAD patients, we examined DLGAP5 mutation rates using five lung adenocarcinoma cohorts. The DLGAP5 mutation rate was generally low, with the highest rate of 3.28% (Figure 7(a)). A total of 16 DLGAP5 mutant sites were identified, including 14 (87.5%) missense mutations, 1 (6.25%) truncating mutation, and 1 (6.25%) splice mutation (Figure 7(b)). Interestingly, the DLGAP5-mutant group had a higher probability of tumor mutation burden (TMB) (Figure 7(c)), a quantifiable biomarker for immune checkpoint blockade (ICB) selection. However, there was no statistical difference in overall survival between the DLGAP5-mutated and DLGAP5-unmutated groups of LUAD patients (Figure 7(d)).

The DNA methylation levels of DLGAP5 were assessed using the MethSurv tool, and six methylated CpG sites were identified. Among them, cg23678254 had the highest level of DNA methylation and was associated with poor prognosis in LUAD patients (Figures 7(e)–7(g)).

### 3.6. Relationship between DLGAP5 Expression and the Clinicopathological Characteristics of LUAD Patients

**Figure 8:**
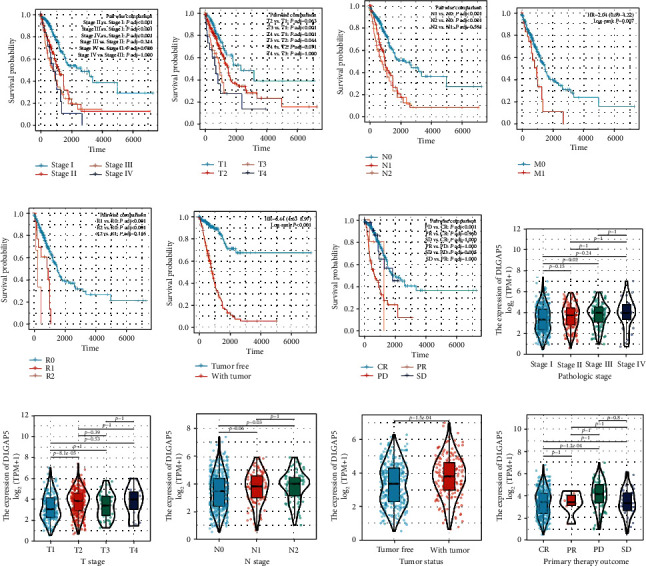
Association of clinical pathological characteristics with prognosis and DLGAP5 expression. The Kaplan-Meier curves showed the overall survival of LUAD patients in relation to pathologic stage (a), T stage (b), N stage (c), M stage (d), residual tumor (e), and tumor status (f). DLGAP5 was differentially expressed in pathologic stage (h), T stage (i), N stage (j), tumor status (k), and primary therapy outcome (l). CR: complete remission/response; PD: progressive disease; PR: partial remission/response; SD: stable disease.

First, we evaluated the relationship between clinicopathological characteristics and the prognosis of LUAD patients. The results showed that the higher the TNM stage of LUAD patients, the lower the survival rate is, such as pathologic stages II and III and IV vs. stage I, T3 and T4 vs. T1, N1 and N2 vs. N1, and M1 vs. M0 (Figures 8(a)–8(d)). In addition, patients with residual tumors R1 and R2 had a higher risk of death compared to R0 (Figure 8(e)). Survival rates were significantly lower in patients with tumors than in tumor-free patients (Figure 8(f)). Finally, the patient's treatment outcome also affected the survival rate. Among them, patients with progressive disease (PD) had a lower survival rate than those with complete remission/response (CR) and stable disease (SD) (Figure 8(g)).

As shown in Figures 8(h)–8(l), increased DLGAP5 expression was significantly correlated with pathologic stage (stage III vs. stage I, *P* = 0.02), T stage (T2 vs. T1, *P* < 0.001), N stage (N2 vs. N0, *P* = 0.03), tumor status (with tumor vs. tumor free, *P* < 0.001), and primary therapy outcome (PD vs. CR, *P* < 0.001). Thus, these results indicated that high expression of DLGAP5 was linked to tumor progression and treatment resistance in LUAD patients.

### 3.7. Prognostic Value and Predictive Efficacy of DLGAP5 in LUAD

**Figure 9:**
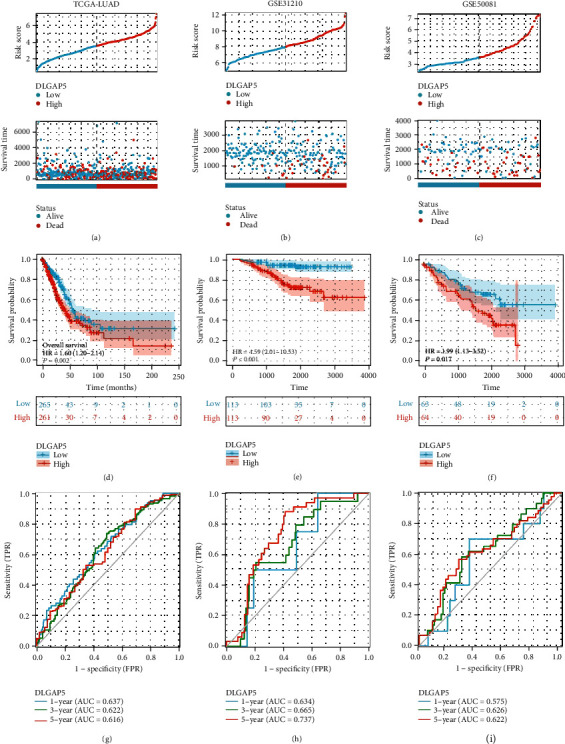
Prognostic value of DLGAP5 in LUAD. (a–c) Scatter plots were performed to represent the survival status and time of LUAD patients in the DLGAP5-high and DLGAP5-low groups. (d–f) The Kaplan-Meier curves were employed to exhibit the effect of DLGAP5 expression on the overall survival of LUAD patients. (g–i) Time-dependent ROCs of DLGAP5 were employed to predict 1-, 3-, and 5-year overall survival of LUAD patients.

To further clarify the prognostic value of DLGAP5, we utilized the TCGA-LUAD, GSE31210, and GSE50081 cohorts for validation. First, scatter plots were performed to roughly estimate the number of deaths and survival times of LUAD patients. The result was that more LUAD patients died in the DLGAP5-high group (Figures 9(a)–9(c)). Next, the Kaplan-Meier analysis showed that LUAD patients in the DLGAP5-high group had a worse prognosis than the DLGAP5-low group (Figures 9(d)–9(f)). In addition, time-dependent ROC curves for DLGAP5 were established to predict 1-, 3-, and 5-year survival in patients with LUAD. All AUC values for predicting 3- and 5-year survival were above 0.6, which was considered appropriate for prediction (Figures 9(g)–9(i)).

**Figure 10:**
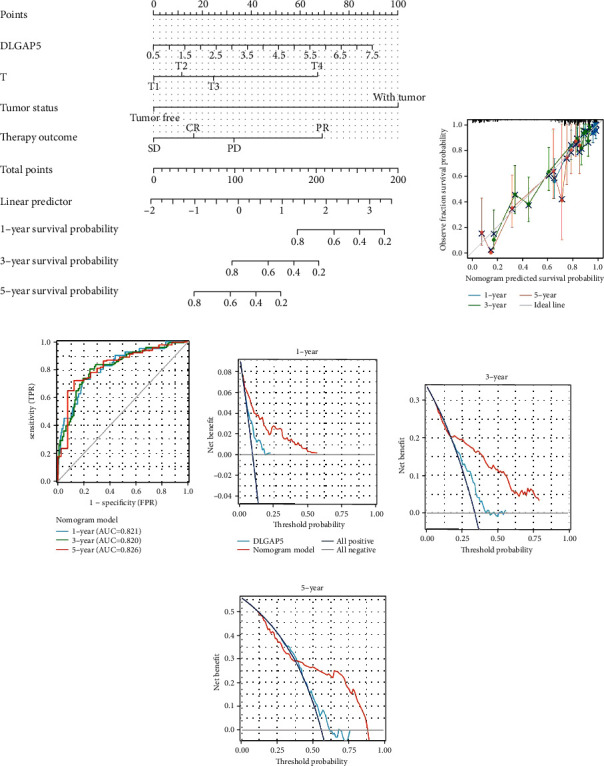
Construction and evaluation of the nomogram model using TCGA-LUAD cohort. (a) DLGAP5 expression, T-stage, tumor status, and therapy outcome were combined to construct a nomogram model. (b) The calibration curves of this nomogram model. Time-dependent ROC (c) and DCA curves (d–f) of the nomogram model predicted the overall survival of LUAD patients at 1, 3, and 5 years, respectively.

As shown in Table 3, we performed univariate and multivariate Cox regression analyses utilizing partial clinicopathological data and DLGAP5 expression in LUAD patients, which further confirmed that DLGAP5 was indeed an independent prognostic risk factor for patients with LUAD. Subsequently, DLGAP5 expression, T stage, tumor status, and therapy outcome were collectively constructed as a nomogram (Figure 10(a)). To evaluate the predictive efficiency of this nomogram, we calculated this model's concordance index (C-index) as 0.777 (95% CI: 0.754-0.800) and plotted the calibration curve (Figure 10(b)). Furthermore, time-dependent ROC curves showed AUC values of 0.821, 0.820, and 0.826 for predicting 1-, 3-, and 5-year OS survival in LUAD patients, respectively (Figure 10(c)). The DCA curves further confirmed that this nomogram model exhibited more promising clinical applications than DLGAP5 expression alone in predicting 1-, 3-, and 5-year overall survival in LUAD patients (Figures 10(d)–10(f)).

## 4. Discussion

In recent years, DLGAP5 has been reported to have a dominant role as an oncogene in a variety of cancers. For instance, DLGAP5 expression was upregulated in various cancers and related to poor prognosis, including endometrial, glioma, bladder, and breast cancers [23, 32–34]. DLGAP5 knockdown resulted in dramatically reduced proliferative and invasive potential in colorectal, clear cell renal cell carcinoma, and hepatocellular carcinoma [15–17]. Interestingly, the detection of DLGAP5 mRNA in urine was a valuable noninvasive test for early diagnosis of bladder cancer and bloodstream bladder cancer, which improved the sensitivity of urine cytology by up to 91% [35]. Consistent with these results, DLGAP5 expression was upregulated in almost all cancers, except LAML. Furthermore, DLGAP5 knockdown significantly inhibited the proliferation and colony formation of lung cancer cells. Collectively, these data strongly indicated a critical role for DLGAP5 in tumorigenesis and progression.

DLGAP5, as a cell cycle regulatory protein, is an essential component of the mitotic apparatus that colocates with the spindle and controls its stability and dynamics [36, 37]. However, tumor cells exploit the property that DLGAP5 can regulate the cell cycle to promote proliferation. For instance, downregulation of DLGAP5 expression suppressed the proliferation and induced cell cycle arrest of ovarian and breast cancer cells [19, 20]. According to the results of our RNA sequencing and enrichment analysis, DLGAP5-related genes were located in the chromosomal and centromeric regions and were primarily engaged in the regulation of the cell cycle. The knockdown of DLGAP5 resulted in cell cycle G1 arrest in lung cancer cells A549 and H1975. The underlying cause of tumor formation is uncontrolled cell division, leading to unlimited proliferation. Therefore, the regulation of the cell cycle becomes a crossroads in tumorigenesis or tumor suppression [23]. Overall, these results showed that DLGAP5 played a critical role in maintaining cellular integrity and determining cell fate.

Cancer development and progression are accompanied by alterations in the surrounding stroma [38]. Tumor infiltrating lymphocytes (TILs), an important component of the stromal cells, have been shown to contribute to tumor progression in the TME [39, 40]. It has been shown that DLGAP5 can activate interleukin-6/Janus kinase 2/signal transducer and activator of transcription 3 (IL-6/JAK2/STAT3) signaling pathway thereby promoting the proliferation and invasion of osteosarcoma cells [41]. Our results clarified that DLGAP5 was implicated in the cytokine-cytokine receptor pathway and was positively associated with Th2 cells. Th2 cells in TME were associated with the progression of lung, breast, cervical, and colorectal cancers [31, 42–44]. Specifically, Th2 cells can produce a variety of cytokines, including IL-4, IL-5, IL-6, IL-9, IL-10, and IL-13 [45]. Among them, IL-5 promotes metastasis via recruitment of sentinel eosinophils that produce CCL22, which recruits regulatory T cells (Treg) to the lung. In the early stages of metastasis, Treg created a protumorigenic microenvironment [46]. In addition, Th2 cells also secrete proangiogenic factors that accelerate uncontrolled angiogenesis and promote vascular immaturity [47]. Moreover, high expression of DLGAP5 reduced the infiltration of various immune cells that exert antitumor effects, such as CD8 T cells, B cells, and NK cells. In brief, DLGAP5 can affect patient prognosis or treatment outcome by reshaping the tumor microenvironment.

As mentioned previously, increased DLGAP5 expression contributed to the resistance of prostate cancer and hepatocellular carcinoma cells to *γ*-radiation and cisplatin, respectively [21, 22]. However, there are no relevant reports on the association of DLGAP5 with immunotherapy. Over the past decade, tremendous progression has been made in the treatment of cancer through immunotherapy, and blocking the immune checkpoint pathway is the most promising strategy for antitumor immunity [48]. Currently, the PD-1/PD-L1 pathway is common clinical immunotherapy targets, and multiple therapeutic antibodies have been approved [49, 50]. However, only a fraction of cancer patients benefit from checkpoint inhibitors [51]. Therefore, it is essential to figure out the mechanisms of immune checkpoints as much as possible. From our results, the expression of PD-1 and PD-L1 was higher in the DLGAP5-high group compared to the DLGAP5-low group. Notably, LUAD patients with high DLGAP5 expression have a poorer response to immunotherapy. Therefore, DLGAP5 can be considered as an indicator to predict the clinical response to immunotherapy.

Although these results broaden our understanding of DLGAP5 in LUAD, there are still some limitations. For example, we utilized the TCGA-LUAD cohort, in which information on some patients is incomplete and the result needs to be further validated with clinical samples. The second is that DLGAP5 serves as an oncogene in LUAD, and knocking it down significantly inhibits cell proliferation *in vitro*. Under physiological conditions, however, DLGAP5 holds a crucial role in maintaining cell cycle stability and proliferation of the female endometrial stroma. Therefore, gene editing mouse models are needed to further comprehensively investigate the function of DLGAP5 *in vivo*.

Overall, we systematically reported the potential function of DLGAP5 in LUAD in this study. DLGAP5 was highly expressed in LUAD tissues and promoted the proliferation of lung cancer cells through regulation of cell cycle. Furthermore, DLGAP5 was related to multiple immune cells in TME and could predict prognosis and response to immunotherapy in LUAD patients. Importantly, we constructed a clinical prognostic model based on DLGAP5 expression, which could effectively predict the probability of 1-, 3-, and 5-year OS for LUAD patients.

## Figures and Tables

**Table 1:** Details of the seven lung adenocarcinoma datasets in the GEO database used in this study.

GEO	Tissue	Platform	Tumor	Normal
GSE7670	Lung adenocarcinoma	GPL96	26	26
GSE43458	Lung adenocarcinoma	GPL6244	80	30
GSE116959	Lung adenocarcinoma	GPL17077	57	11
GSE140797	Lung adenocarcinoma	GPL13497	7	7
GSE31210	Lung adenocarcinoma	GPL570	226	NA
GSE50081	Lung adenocarcinoma	GPL570	127	NA
GSE143423	Lung adenocarcinoma	GPL20795	3	NA

**Table 2:** Correlation of DLGAP5 expression with immune cells in TME.

Immune cells	Cor/*P* value	Immune cells	Cor/*P* value	Immune cells	Cor/*P* value
Mast cells	-0.458/^∗∗∗^	NK CD56bright cells	-0.164/^∗∗∗^	Neutrophils	-0.007/ns
Eosinophils	-0.332/^∗∗∗^	Natural killer cells	-0.156/^∗∗∗^	Type 1 helper cells	0.014/ns
Immature dendritic cells	-0.300/^∗∗∗^	B cells	-0.109/^∗^	Regulatory T cells	0.085/^∗^
Dendritic cells	-0.258/^∗∗∗^	Macrophages	-0.089/^∗^	Activated dendritic cells	0.149/^∗∗∗^
T follicular helper cells	-0.252/^∗∗∗^	T cells	-0.061/ns	T helper cells	0.214/^∗∗∗^
CD8 T cells	-0.234/^∗∗∗^	Central memory T cells	-0.052/ns	NK CD56dim cells	0.246/^∗∗∗^
Plasmacytoid dendritic cells	-0.197/^∗∗∗^	Effector memory T cells	-0.024/ns	Gamma delta T cells	0.274/^∗∗∗^
Type 17 helper cells	-0.194/^∗∗∗^	Cytotoxic cells	-0.013/ns	Type 2 helper cells	0.831/^∗∗∗^

Significance codes: ns: not significant. ^∗^*P* < 0.05, ^∗∗^*P* < 0.01, and ^∗∗∗^*P* < 0.001.

**Table 3:** Univariate and multivariate Cox regression analyses of clinical data of LUAD patients (OS).

Characteristics	Total (*N*)	Univariate Cox analysis		Multivariate Cox analysis	
		Hazard ratio (95% CI)	*P* value	Hazard ratio (95% CI)	*P* value
DLGAP5	535	1.299 (1.162-1.453)	<0.001	1.257 (1.025-1.543)	0.028
*Pathologic stage*	527				
Stage I	294	Reference			
Stage II	123	2.305 (1.617-3.284)	<0.001	0.300 (0.105-0.852)	0.024
Stage III	84	3.439 (2.378-4.974)	<0.001	0.471 (0.077-2.878)	0.415
Stage IV	26	3.601 (2.089-6.209)	<0.001	0.486 (0.151-1.563)	0.226
*T stage*	532				
T1	175	Reference			
T2	289	1.578 (1.110-2.242)	0.011	1.212 (0.644-2.280)	0.552
T3	49	2.898 (1.723-4.874)	<0.001	3.587 (1.196-10.758)	0.023
T4	19	3.309 (1.742-6.285)	<0.001	7.812 (1.664-36.679)	0.009
*N stage*	519				
N0	348	Reference			
N1	95	2.300 (1.640-3.224)	<0.001	2.502 (0.935-6.696)	0.068
N2	74	3.054 (2.110-4.421)	<0.001	2.693 (0.548-13.228)	0.222
N3	2	0.000 (0.000-Inf)	0.994	0.000 (0.000-Inf)	0.997
*M stage*	386				
M0	361	Reference			
M1	25	2.056 (1.203-3.514)	0.008		
*Residual tumor*	372				
R0	355	Reference			
R1	13	3.108 (1.620-5.962)	<0.001	0.878 (0.307-2.512)	0.808
R2	4	9.579 (2.980-30.788)	<0.001	0.000 (0.000-Inf)	1.000
*Tumor status*	480				
Tumor free	300	Reference			
With tumor	180	6.576 (4.523-9.560)	<0.001	8.471 (4.606-15.579)	<0.001
*Therapy outcome*	446				
CR	332	Reference			
SD	37	1.070 (0.538-2.126)	0.848	0.895 (0.310-2.588)	0.838
PD	71	3.501 (2.445-5.011)	<0.001	2.897 (1.554-5.400)	<0.001
PR	6	2.426 (0.595-9.893)	0.216	14.889 (3.193-69.422)	<0.001
*Gender*	535				
Male	249	Reference			
Female	286	0.933 (0.702-1.239)	0.631		

CR: complete remission/response; PD: progressive disease; PR: partial remission/response; SD: stable disease.

## Data Availability

The data used to support the findings of this study are included within the article.
